# A case of *Staphylococcus epidermidis* osteomyelitis in the absence of spine hardware

**DOI:** 10.1016/j.idcr.2024.e01928

**Published:** 2024-01-11

**Authors:** Erin Coonahan, Bita Shahrvini, Morgan Birabaharan, Nikdokht Farid, Annie Cowell

**Affiliations:** a School of Medicine, University of California San Diego (UCSD), 9500 Gilman Drive, La Jolla, CA 92093, USA; b Division of Infectious Diseases and Global Public Health, UC San Diego Health Department of Medicine, San Diego, California, USA; c Department of Radiology, University of California San Diego, La Jolla, CA, USA

**Keywords:** Osteomyelitis, *Staphylococcus epidermidis*, Endocarditis, Magnetic Resonance Imaging (MRI)

## Abstract

*Staphylococcus epidermidis* is a typically indolent pathogen that is often considered a blood culture contaminant. It is a rare and unexpected cause of osteomyelitis, especially in the absence of recent surgical intervention or orthopedic implants. We highlight a case in which a 90-year-old Caucasian male with no recent spine surgery was found to have osteomyelitis of the lumbar spine and repeat positive blood cultures for methicillin resistant *Staphylococcus epidermidis* (MRSE). Further investigation revealed a history of mitral valve replacement and a new diagnosis of endocarditis leading to persistent bacteremia and seeding of his lumbar vertebrae. This case demonstrates that *S. epidermidis* can cause vertebral osteomyelitis resulting in severe complications that are more similar to highly pathogenic bacteria. We describe the steps to diagnosing this chronic undetected infection and related comorbidities.

## Introduction

Rates of vertebral osteomyelitis have increased in the United States in recent decades, almost doubling from 1998–2013 [1]. Increasing rates have also been reported in other countries with aging populations and advanced age is one of the most important risk factors for disease [2], [3]. Osteomyelitis of the vertebrae can be challenging to recognize, with a typical delay in diagnosis of greater than 45 days from symptom onset and as many as one-third of patients initially receiving a misdiagnosis [4], [5], [6]. Lagging detection is due in large part to the nonspecific presentation [3]. The most common presenting symptoms are back pain and elevated inflammatory markers (erythrocyte sedimentation rate (ESR) and C-reactive protein (CRP)). Notably, fever is absent in as many as half of all presentations [4]. The most commonly identified sources of infection include urinary tract, skin and soft tissue, and catheter relation blood stream infections [7].

*Staphylococcus aureus* is the most common cause of vertebral osteomyelitis, accounting for over half of all cases [8], [9]. While still the most likely causative organism, *S. aureus* is significantly less common in patients over 65 compared to patients under 65. Older patients reportedly have higher rates of infection with streptococcal species and gram-negative rods such as *Escherichia coli* [7]. *Staphylococcus epidermidis* accounts for roughly 5–20 % of cases, almost exclusively following spine surgery and implantation of spine hardware [9], [10], [11]. Here we present a patient without recent surgical intervention diagnosed with vertebral osteomyelitis on imaging and positive blood cultures for methicillin resistant *Staphylococcus epidermidis* (MRSE).

### Case report

A 90-year-old male presented to the emergency department for 3 months of progressive weakness and chronic back pain. Medical history included bladder cancer, severe mitral regurgitation status post mitral valve replacement (6 months prior), a recent stroke (3 months prior) with residual left sided weakness and hearing loss, and a recent diagnosis of type two diabetes mellitus (HbA1c 6.7 %).

In the ED, the patient was found to be severely anemic (Hgb 5.4 [ref range: 14–18 g/dL]) and hyperglycemic (Glucose 584 [ref range: 80–140 mg/dL]). His vital signs were stable and within normal limits, and physical exam was remarkable for a 2/6 systolic murmur and tenderness to palpation over the lumbosacral spine and paraspinal muscles without associated focal neurological deficits.

**Fig. 1:**
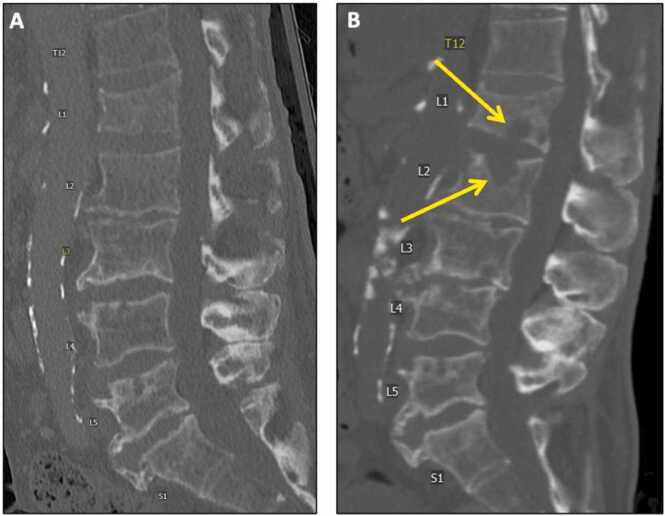
Discitis-osteomyelitis changes on CT. Comparing to prior CT L-spine from March 2022 (A), a CT L-spine from August 2022 (B) shows new prominent erosive endplate changes at L1-L2 (arrows) and some erosion at L3-L4, concerning for discitis-osteomyelitis.

**Fig. 2:**
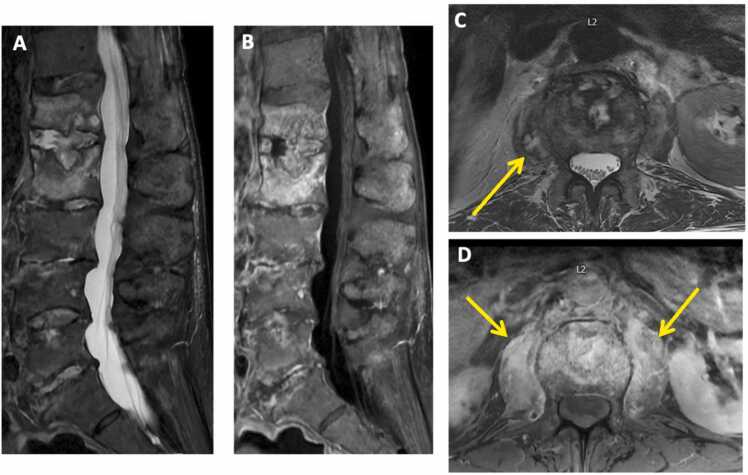
MRI confirms discitis-osteomyelitis changes observed on CT. Sagittal STIR (A) and post-contrast images (B) demonstrate prominent edema and enhancement of the L1 and L2 vertebral bodies and within the L1-L2 intervertebral disc, compatible with discitis-osteomyelitis. There is also mild edema and enhancement of the L3-L4 vertebral endplates, which in conjunction with new erosive endplate changes seen on the CT, are suggestive of early discitis-osteomyelitis at this level. C. Axial STIR image demonstrates a small heterogeneous collection in the right psoas muscle. D. There is no definite rim-enhancement on the Axial T1 post-contrast image. Therefore, this is most compatible with a phlegmon, rather than a frank abscess. Axial T1 post-contrast image also demonstrates diffuse enhancement of the bilateral psoas muscles compatible with inflammatory change.

Given his severe anemia and back pain, a CT abdomen/pelvis was ordered to assess for possible retroperitoneal bleed. Incidentally, CT revealed discitis-osteomyelitis changes involving L1–2 and to a lesser extent L3–4 (Fig. 1). MRI confirmed these findings and exposed a small paraspinal phlegmon measuring 1.4 × 1.3 cm determined to be inaccessible by interventional radiology for sampling (Fig. 2). These imaging findings and a rising CRP (from 6.14 on admission to 12.11) prompted further workup to establish a possible infectious source of hematologic translocation causing vertebral osteomyelitis.

Blood cultures for this patient were positive for methicillin resistant *S. epidermidis* (MRSE), typically considered a blood culture contaminant especially in the absence of indwelling catheters or surgical hardware. However, the presence of a prosthetic mitral valve along with serial blood cultures remaining positive until day 10 of admission, raised suspicion that the *S. epidermidis* was a true causative organism. Given the patient’s history of mitral valve replacement and systolic murmur on physical exam, echocardiography was obtained to assess for endocarditis. No vegetations were visualized on initial transthoracic echocardiogram. However, high clinical suspicion for endocarditis prompted further evaluation with a transesophageal echocardiogram which demonstrated perivalvular dehiscence and leaking with worsening mitral regurgitation concerning for probable chronic prosthetic valve endocarditis (PVE). Thus, the vertebral osteomyelitis was likely a complication of PVE with MRSE.

Ultimately, given the patient’s age and comorbidities, he was not a candidate for surgical management. After one week of vancomycin with persistently positive blood cultures, the patient was switched to daptomycin, ceftaroline, and gentamicin for salvage therapy extrapolated from successful treatment of methicillin resistant *Staphylococcus aureus* persistent bacteremia [12]. After approximately one additional week on this regimen the patient developed acute renal injury and was subsequently switched to intravenous daptomycin for ease of dosing and decreased risk for renal injury and rifampin. He was discharged with an eight-week total course of antibiotics but unfortunately passed away at a skilled nursing facility several weeks after hospital discharge, further follow-up information was not available.

## Discussion

As vertebral osteomyelitis due to *S. epidermidis* is unusual in the absence of spine hardware, workup in this case included investigation for a persistent cause of intravascular infection. Several recent studies detected infective endocarditis (IE) in 10–15 % of patients with vertebral osteomyelitis. [13], [14] Other studies have reported coinfection rates as high as 33 % with variation due to patient demographics (higher rates of IE were reported in older and male patients) and sensitivity of imaging modalities. [15] Infective endocarditis (IE) should be considered in all cases of vertebral osteomyelitis especially in elderly patients and those with a history of valvulopathy. One recent study reported that concomitant IE was found in 38.4 % of patients with vertebral osteomyelitis > 65 years old. [7] Given the similar clinical presentation of back pain, fever, fatigue, and elevated inflammatory markers, it may be difficult to assess likelihood of IE in patients with vertebral osteomyelitis and appropriate imaging should be obtained where there is reasonable suspicion.

Here, we present a patient with chronic back pain, diagnosed with osteomyelitis on MRI that prompted an investigation revealing significant morbidity caused by a typically indolent organism. Further chart review revealed a positive *S. epidermidis* urine culture in the absence of urinary instrumentation during his admission 3 months prior for stroke. This result raises concern for a bloodstream source of infection since that time, indicating prolonged chronicity of infection. Infection of his prosthetic mitral valve likely led to seeding of his spine due to septic emboli and was also a probable cause of his cardioembolic stroke 3 months earlier. Furthermore, his chronic, severe infection was hypothesized to be driving a hyperadrenergic state leading to his unusual diabetes diagnosis at age 90 and severe hyperglycemia. *S. epidermidis* is rarely considered to be pathogenic but this case provides an example where it resulted in significant morbidity and highlights that it should not be disregarded, especially in individuals with intravascular prosthetic devices and persistently positive blood cultures.

For treatment of native vertebral osteomyelitis, Infectious Diseases Society of America (IDSA) recommends 6 weeks of parenteral or highly bioavailable oral antimicrobial therapy. For oxacillin susceptible strains, nafcillin, oxacillin, cefazolin, or ceftriaxone should be used. For oxacillin resistant strains, vancomycin is first line treatment [4]. PVE caused by *S. epidermidis* is usually methicillin resistant, especially within 1 year after surgery. However, for susceptible strains, treatment of *S. epidermidis* PVE includes nafcillin or oxacillin plus rifampin plus gentamicin. For methicillin resistant strains, as in this patient, treatment includes vancomycin plus rifampin plus gentamicin. Duration of treatment is typically at least 6 weeks with gentamicin given for the first 2 weeks only. [16].

## Ethical approval

Our institution does not require ethical approval for reporting individual cases or case series.

## Conflicts of interest

The authors declare that there is no conflict of interest.

## Informed consent

Verbal informed consent was obtained from a legally authorized patient representative. Patient information is anonymized.

## Funding

The authors received no financial support for the research, authorship, and/or publication of this article.

## CRediT authorship contribution statement

All authors had access to the data and a role in writing this manuscript. EC: Writing – Original Draft, Visualization, BS: Writing – Original Draft, MB: Conceptualization, NF: Visualization, Writing – Reviewing and Editing, AC: Conceptualization, Writing - Reviewing and Editing.

## Declaration of Competing Interest

The authors declare that they have no known competing financial interests or personal relationships that could have appeared to influence the work reported in this paper.
